# The role of human mast cells in allergy and asthma

**DOI:** 10.1080/21655979.2022.2044278

**Published:** 2022-03-10

**Authors:** Ghalya H Banafea, Sherin Bakhashab, Huda F Alshaibi, Peter Natesan Pushparaj, Mahmood Rasool

**Affiliations:** a Biochemistry Department, King Abdulaziz University, Jeddah, Saudi Arabia; b Department of Medical Laboratory Technology, Faculty of Applied Medical Sciences, King Abdulaziz University, Jeddah, Saudi Arabia; c Center of Excellence in Genomic Medicine Research, Faculty of Applied Medical Sciences, King Abdulaziz University, Jeddah, Saudi Arabia

**Keywords:** Mast cells, cytokines, chemokines, histamine, proteases, inflammation, asthma, allergy

## Abstract

Mast cells are tissue-inhabiting cells that play an important role in inflammatory diseases of the airway tract. Mast cells arise in the bone marrow as progenitor cells and complete their differentiation in tissues exposed to the external environment, such as the skin and respiratory tract, and are among the first to respond to bacterial and parasitic infections. Mast cells express a variety of receptors that enable them to respond to a wide range of stimulants, including the high-affinity FcεRI receptor. Upon initial contact with an antigen, mast cells are sensitized with IgE to recognize the allergen upon further contact. FcεRI-activated mast cells are known to release histamine and proteases that contribute to asthma symptoms. They release a variety of cytokines and lipid mediators that contribute to immune cell accumulation and tissue remodeling in asthma. Mast cell mediators trigger inflammation and also have a protective effect. This review aims to update the existing knowledge on the mediators released by human FcεRI-activated mast cells, and to unravel their pathological and protective roles in asthma and allergy. In addition, we highlight other diseases that arise from mast cell dysfunction, the therapeutic approaches used to address them, and fill the gaps in our current knowledge. Mast cell mediators not only trigger inflammation but may also have a protective effect. Given the differences between human and animal mast cells, this review focuses on the mediators released by human FcεRI-activated mast cells and the role they play in asthma and allergy.

## Graphical Abstract

**Figure:**
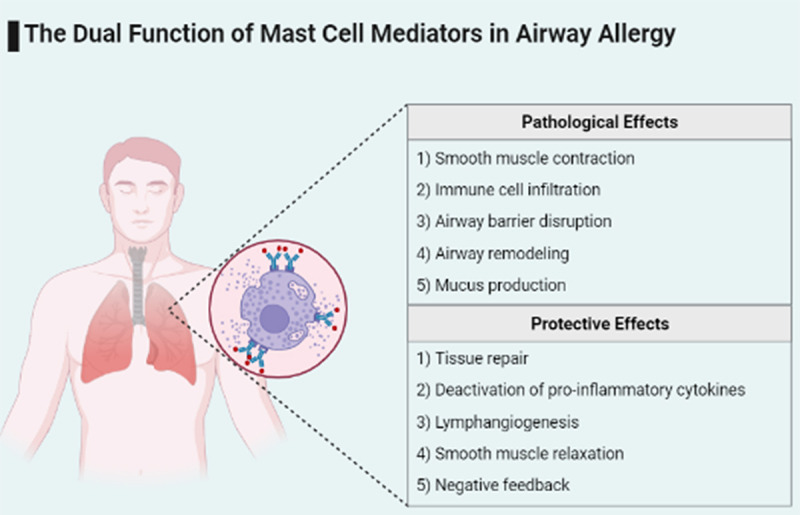

## Introduction

Allergic asthma, also known as airway hypersensitivity, is a chronic inflammatory disease of the lower airways characterized by airway smooth muscle contraction, increased mucus secretion, infiltration of the submucosa, and airway smooth muscle by immune cells, and airway remodeling. According to the European Academy of Allergy and Clinical Immunology [1], allergic asthma is the most common asthma phenotype in children and adults as of 2021, causing over 90% and 50% of all asthma cases, respectively.

**Figure 1.**
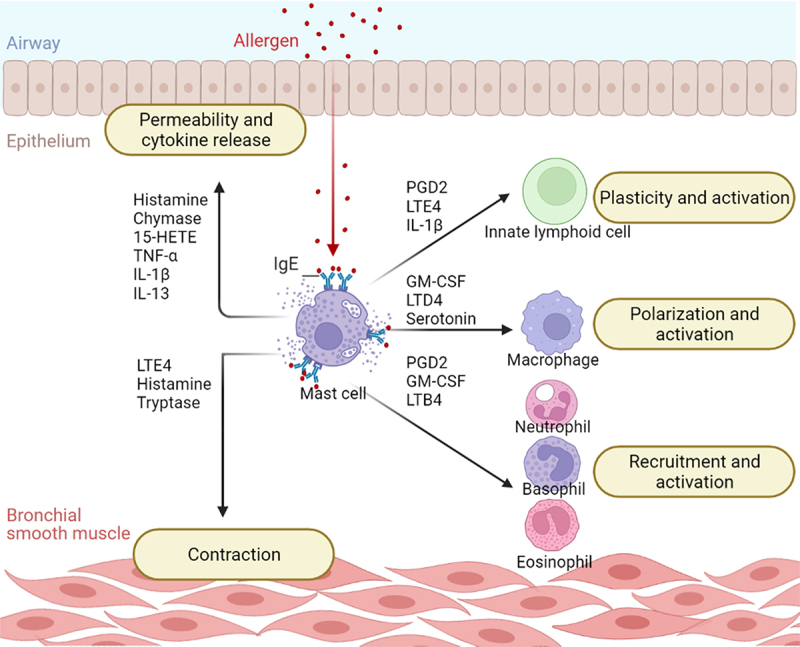
**Mast cells exacerbate airway inflammation by communicating with other cells**. After IgE cross-linking, mast cells release a variety of mediators that drive inflammation by activating other cells. Mast cells contribute to asthma symptoms by triggering bronchoconstriction and altering the permeability of the bronchial barrier. They also recruit other immune cells and promote their proliferation and activation. Key: 15-HETE, 15-Hydroxyeicosatetraenoic Acid; GM-CSF, Granulocyte-Macrophage Colony Stimulating Factor; IL-13, Interleukin-13; IL-1β, Interleukin-1β; LTB4, Leukotriene B4; LTD4, Leukotriene D4; LTE4, Leukotriene E4; PGD2, Prostaglandin D2; TNF-α, Tumor Necrosis Factor α.

Cell types that accumulate in the airways of asthma patients include mast cells [2,3]. Mast cells belong to the myeloid lineage and arise from the hematopoietic stem cells of the bone marrow, migrate through the blood as progenitor cells, and complete their differentiation at the target tissue as large, granular, tissue-inhabiting cells [4]. They are multifunctional cells capable of recognizing pathogen-associated molecular patterns via Toll-like receptors, internal signals via cytokine and chemokine receptors, and antigens via IgE-FcεRI cross-linking [5–7]. Because mast cells reside in tissues in contact with the external environment and express a variety of receptors, they play an essential role in initiating a rapid response to pathogen invasion and tissue damage. In response to various stimulants such as allergens, cytokines, or pathogen-associated molecular patterns mast cells release several mediators that can recruit and enhance the function of other immune cells such as T cells and dendritic cells creating the link between innate and acquired immunity. In addition, mast cells participate in antigen presentation and promote wound healing and tissue repair, models of mast cell deficiency exhibit slower clearance of bacterial and parasitic infections, however drug-induced mast cell deficiency in human has not reported significant side effects on tissue homeostasis [8–10]. In contrast abnormally increased numbers of infiltrating mast cells are observed in mastocytosis, whereas mast cell activation have also been implicated in inflammatory conditions such as inflammatory bowel disease, chronic skin inflammation, and allergies [11,12]. Patients with allergic asthma suffer dyspnea, wheezing and fatigue [1], these symptoms are driven by FcεRI-activated mast cells which release a range of pro-inflammatory mediators thus recruiting other immune cells, inducing bronchoconstriction and airway remodeling (Figure 1).

## Mast cell activation through IgE-FcεRI cross-linking

Mast cell degranulation is a hallmark of allergy that can be triggered by several factors. There are two main pathways to trigger mast cell degranulation: The first, is via stimulation of Mas-Related G Protein-Coupled Receptor-X2 (MRGPRX2), independently of IgE [13]; while the second, and better-studied pathway involves the interaction between the allergen-IgE complex and the high-affinity IgE receptor (FcεRI) on the surface of mast cells.

IgE is infamous for mediating allergic reactions, it belongs to the immunoglobulin (Ig; or antibody) family, which is critical for antigen entry into immune cells. Immunoglobulins are produced by plasma cells and can interact with different cell types depending on the type of Fc chain. There are 5 types of Fc chains and thus 5 classes of immunoglobulins: IgA, IgD, IgE, IgG, and IgM, while the specificity of the target cell is determined by the Fc chain, antigen recognition occurs via the variable region: Fab, lymphocytes produce different Fab regions via the process of variable, diverse, and joining (VDJ) recombination, which allows the body to respond to different antigens. Upon initial exposure to an allergen, mast cell sensitization occurs through the interaction between specific epitope in IgE and the allergen. This process allows the mast cells to respond rapidly to the allergen upon subsequent exposure [14]. Increased numbers of sensitized mast cells were observed in alveoli of asthma patients in comparison to healthy controls [2].

## Mast cell mediators in allergy and asthma

Activation of mast cells in response to IgE-FcεRI stimulation leads to two important events: a) the release of proteases, proteoglycans, and neuropeptides stored in the granules (Table 1) due to the increase in intracellular calcium levels mediated by phospholipase C gamma 1 (PLC-γ1) phosphorylation [15] and b) the secretion of *de novo* synthesized lipid mediators and cytokines (Table 2) mediated by the activation of nuclear factor kappa B (NF-κB), a major transcription factor that enhances the expression of numerous genes related to inflammation; all these factors act together to control inflammation [16].

**Table 1.** Granule-stored mediators released by mast cells upon FcεRI activation

	Mediator	Effect	Reference
**Monoamines**	Histamine	Induces smooth muscle contraction	[17,18,27,128]
**Proteoglycans**			
	Heparin	Reduce mast cells’ production of pro-inflammatory cytokines.	[27,28]
	Chondroitin sulfates	Reduce mast cells’ production of pro-inflammatory cytokines.	[27,28]
**Proteases**	Chymase	Promotes airway remodeling and chemotaxis.	[58–60]
	Tryptase	Promotes airway smooth muscle contraction and facilitates tissue repair.	[61,62]
	Carboxypeptidase 3	Promotes vasodilation.	[56,66]
	Cathepsin G	Regulates the activity of cytokines.	[65,67]
**Neuropeptides**	PACAP	Induces mast cell degranulation and smooth muscle relaxation.	[74–76]
	Serotonin	Promotes Bronchial Smooth Muscle Cell proliferation.	[68,71,73]

**Table 2.** *De Novo* Synthesized mediators released by Mast cells upon FcεRI activation

	Mediator	Effect	Reference
**Lipid mediators**	**Prostaglandins**		
	PGD2	Eosinophil maturation, ILC2 recruitment, production of pro-inflammatory cytokines.	[43,103]
	PGE2	Reduces production of inflammatory cytokine and mast cell degranulation	[38,39]
	**Leukotrienes**		
	LTB4	Mast cell recruitment	[48]
	LTC4	Induces production of growth factors by bronchial smooth muscle cells and monocytes	[43,49]
	LTD4	Upregulates the production of Inflammatory cytokines by mast cells	[53]
	LTE4	Promotes bronchoconstriction and recruitment of immune cells	[50]
**Cytokines**	IL-1β	Increases mucus production by bronchial epithelial cells and ILC3 plasticity.	[80–82,129]
	IL-6	Enhances mast cell proliferation and activation.	[79,84,85,88,129]
	IL-13	Increase mucus production, tissue repair and histamine signaling.	[36,83,130]
	TNF-α	Increase the production of pro-inflammatory cytokines and proliferation of airway epithelium	[78,103,131]
	GM-CSF	Enhance the granulocytes’ growth and macrophage polarization.	[110,111,121]
**Chemokines**	CCL1	Increase ILC2 expansion, growth, and survival	[91,98]
	CCL2 (MCP-1)	Increase neutrophil and monocyte recruitment.	[94,96,131]
	CCL3	Increase monocyte recruitment	[91,100]
	CCL4	Increase eosinophil recruitment	[92,93]
	CCL5	Essential for monocyte recruitment	[92,99]
	CCL7	Increases neutrophil recruitment	[92,94]
	CXCL1	Increases neutrophil recruitment	[45,92]
	CXCL3	Stimulates airway smooth muscle cell migration	[92,101]
	CXCL8 (IL-8)	Increases the recruitment of CD4 + T-cells and neutrophils	[91,92,131]
**Growth Factors**	VEGF-A	Promotes angiogenesis	[102,103]
	VEGF-C	Promotes lymphangiogenesis	[102,108]
	HB-EGF	Fibroblast migration and proliferation	[96,104]

## Histamine

Histamine is the most prominent mediator in allergies. It is a monoamine released by mast cell granules in response to FcεRI stimulation. Histamine receptors are divided into 4 main types: H1R, H2R, H3R, and H4R that are widely distributed in various tissues, including the respiratory tract and immune cells, allowing histamine to exert multiple effects during inflammation. Histamine released by mast cells increases the airway epithelial barrier permeability and induces smooth muscle contraction by increasing Ca^2+^ uptake and mobilizing cellular Ca^2+^ stores. These effects are exerted through the signaling of H1R expressed by epithelial and smooth muscle cells [17–19]. H1R receptor signaling also increases the production of prostaglandins, proinflammatory cytokines, and chemotactic factors causing immune cell accumulation. In addition, histamine increases the expression of its receptors and promotes the activation of the NF-κB pathway [20–22]. Moreover, histamine acts synergistically with IL-4 via the H2R receptor to increase the expression of chemokines in macrophages, but H2R signaling also exerts a protective effect in T-cells as it stimulates their production of the anti-inflammatory cytokine IL-10 [23]. The H3R receptor was thought to be expressed primarily in the central nervous system, whilst Kang and colleagues had also detected its expression in the epithelial cells and submucosal glands of human nasal tissues, but it is still unknown whether it plays a role in allergies in the airways [24]. In addition, H4R is also expressed on mast cells and promotes their accumulation in the lungs. H4R signaling leads to the activation of extracellular signal-regulated kinase (ERK) and the nuclear factor of activated T-cells (NFAT), which in turn induce mast cells production of tumor necrosis factor-alpha (TNF-α) and the chemokine IL-8 [25,26]. Thus, histamine produced by mast cells amplifies its influence through H1R and H4R and promotes chronic inflammation.

## Proteoglycans

Mast cells are characterized by their metachromasia, a feature caused by their granules containing proteoglycans such as chondroitin sulfate and heparin which are also released upon activation. These proteoglycans are released along with histamine during mast cell degranulation; however, they have anopposite effect. Chondroitin sulfate is mostly associated with structural functions in the connective tissue while heparin is a major anticoagulant, but both proteoglycans also possess anti-inflammatory properties. Upon mast cell degranulation, these proteoglycans exert anti-inflammatory effect on mast cells by reducing their release of TNF-α and IL-8, possibly by reducing NF-κB activity, thus antagonizing the action of histamine [27,28].

## Prostaglandins

Human mast cells consume arachidonic acid from their phospholipid membrane to synthesize two major groups of lipid mediators: Prostaglandins and leukotrienes.

Oxidation of arachidonic acid by the cyclooxygenase enzymes COX-1/ COX-2 produces prostaglandins (PGs) [29]. PGD2 is produced in mast cells by COX-1 in response to activation by IgE or histamine [19,21,30,31]. It is a potent proinflammatory mediator that is metabolized to produce bioactive metabolites such as DK-PGD_2_, 15-deoxy-∆^12,14^-PGD_2_, and 9α,11β-PGF_2_, amongst others were found to be elevated in asthma patients [32–34]. Airway smooth muscle cells express the PGD2 receptor DP1, signaling through this receptor activates Ras homolog kinase (Rho-kinase) increasing airway smooth muscles’ sensitivity to Ca^2+^ thus augmenting bronchoconstriction [35]. Moreover, PGD2 and its metabolites promote eosinophil maturation directly or indirectly by recruiting innate lymphoid cell type 2 (ILC2) and stimulating their production of IL-5 and IL-13. Therefore, ILC2 exerts a dual effect during airway inflammation, exacerbating inflammation by promoting eosinophil maturation via IL-5 and repairing damaged tissue via IL-13 signaling [32,36].

However, mast cells also express COX-2 which is important for the production of PGE2, which has an anti-inflammatory effect [37]. Mast cells possess the PGE2 receptors EP2, EP3 and EP4. Interaction of PGE2 with EP2 and EP4 suppresses mast cell degranulation by inhibiting phosphorylation of PLCγ1 and ERK1/2, which are critical for calcium influx and degranulation. Therefore, there is an inverse correlation between PGE2 levels and histamine release, thus an individual’s susceptibility to anaphylaxis [38]. ILC2 also expresses EP2 and EP4 receptors, hence PGE2 antagonizes the effect of PGD2 on ILC2. It reduces their proliferation by downregulating the expression of transcription factor GATA binding protein 3 (GATA3) and the IL-2 receptor α essential for ILC2ʹ.s maintenance and function, and downregulating their production of IL-5 and IL-13 [39].

Synthesis of PGs in IgE-activated mast cells by the enzyme COX-1 also produces the by-product 15(S)-hydroxy-5,8,11-cis-13-trans-eicosatetraenoic acid (15-HETE) [40], which is important for the differentiation of mucus-producing airway goblet cells by increasing the expression of the transcription factor Forkhead Box A3 (FOXA3) and stimulating mucus production by increasing the expression of mucin 5AC (Muc5ac). HETE-15 has also been shown to mediate the expression of growth factors in bronchial epithelial cells by activating Signal Transducer And Activator Of Transcription 3 (STAT3) [41,42].

## Leukotrienes

Leukotrienes are the second group of lipid mediators produced by activated mast cells. They are synthesized from arachidonic acid, which is oxidized by lipooxygenase enzymes [29,43,44]. The individual leukotrienes LTB4, LTC4, LTD4, and LTE4 mediate different functions during inflammation (Figure 1); leukotriene B4 (LTB4) is a potent chemoattractant, it recruits neutrophils by acting on BLT_2_ receptor on their surface, and facilitates the recruitment and activation of natural killer cells via BLT_2_ and BLT_1_, respectively [45–47], it also recruits mast cell progenitors to the site of inflammation by acting on BLT_1_ which is only expressed on immature mast cells [48]. LTC4 promotes tissue remodeling and neovascularization by activating the mitogen-activated protein kinase (MAPK) pathway and increasing the expression of growth factors. It also takes part in recruiting ILC2 and induces bronchoconstriction. Interestingly, lung mast cells produce significantly higher amounts of LTC4 in response to IgE stimulation in comparison to skin or connective tissue mast cells [43^,49–52^]. LTD4 increases the production of the pro-inflammatory cytokine TNF-α and the neutrophil and macrophage chemokines IL −8 and C-C Motif Chemokine Ligand 4 (CCL4) by mast cells through the cysteinyl leukotriene receptor 1 (CysLT1R) which leads to the activation of protein kinase C (PKC) and ERK signaling. Surprisingly, these effects are enhanced when combined with PGE2 [53]. Moreover, LTD4 promotes airway remodeling by increasing the expression of growth factors in bronchial smooth muscle cells and monocytes [49]. Finally, LTE4 promotes bronchoconstriction, increases the number of circulating immune cells [50], the migration of ILC2 and cytotoxic T cells, and the production of IL-13, IL-8, granulocyte-macrophage colony-stimulating factor (GM-CSF), and amphiregulin; suggesting that it contributes to the immune cell accumulation and airway remodeling observed in asthma [51,54].

## Mast cell proteases

For decades, mast cells have been classified into 2 types according to the combination of proteases contained in their granules. Under healthy conditions, mast cells located in connective tissue contain tryptase, chymase, cathepsin G and carboxypeptidase A (MCTC), whereas those located in mucosal tissue contain only tryptase (MCT) [55,56]. This is now considered an oversimplification, as mast cells with the same combinations may differ in other characteristics such as size, surface receptors, granulation patterns, and responses depending on their location [43,52,57].

The contrast between mast cell subtypes has also been observed in their distribution pattern in health and disease. In nonasthmatics, MCTC predominates in the pulmonary vessels and pleura while MCT prevails in the bronchi and alveoli [57]. However, two independent studies have shown that MCTC infiltrates the bronchi, epithelium, and submucosa of asthma patients. This trend was also observed with the increase in disease severity, suggesting that chymase may be related to asthma severity [2,3]. Mast cell chymase hinders the growth of the epithelial cells in the bronchi, it also induces morphological changes as these cells elongate and become smaller. Moreover, chymase has been found to enhance bronchial epithelial cell motility; alter the expression of adhesion molecules; induce the expression of the chemokines IL-8, C-X-C motif chemokine ligand 1 (CXCL1), CXCL5, CXCL6, and CCL2. Chymase also induces morphological changes in the airways, degrades fibronectin, and activates the gelatinase MMP2.Thus, chymase plays a key role in airway remodeling and the induction of chemotaxis [58–60].

**Figure 2.**
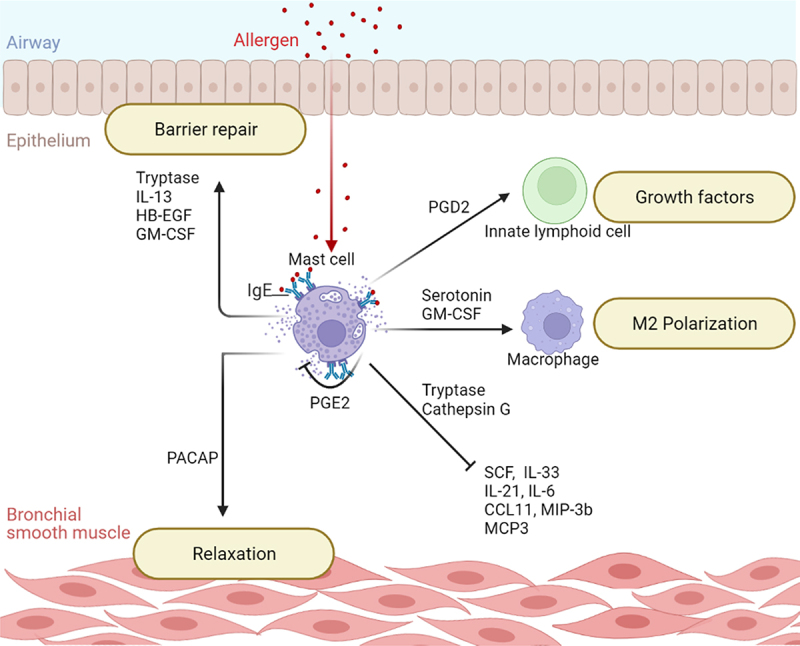
**The protective role of mast cells**. Mast cells regulate their own activation and production of proteases capable of inactivating pro-inflammatory cytokines and keep airway inflammation under control. They are also involved in repairing the airway epithelial barrier by releasing growth factors and promoting anti-inflammatory M2 macrophage polarization. Key: CCL11, C-C Motif Chemokine Ligand 11; GM-CSF, Granulocyte-Macrophage Colony Stimulating Factor; HB-EGF, Heparin-Binding Epidermal Growth Factor; IL-13, Interleukin-13; IL-21, Interleukin-21; IL-33, Interleukin-33; IL-6, Interleukin-6; MCP3, Monocyte Chemotactic Protein 3; MIP-3b, Macrophage Inflammatory Protein 3 beta; PACAP, Pituitary Adenylate Cyclase-Activating Polypeptide; PGD2, Prostaglandin D2; PGE2, Prostaglandin E2; SCF, Stem Cell Factor.

Chymase exhibits potent pro-inflammatory activity, whereas tryptase exerts dual activity, i.e., pro- and anti-inflammatory. Tryptase increases the expression of CCL2 and the pro-inflammatory cytokines IL-6 and IL-22 by epithelial cells; induces airway smooth muscle contraction and fibrinogen degradation, all of which contribute to airway obstruction associated with asthma [58–61]. However, tryptase counteracts the influence of chymase on cell growth by increasing the expression of the proliferative nuclear protein Ki-67 [58,62]. Moreover, it promotes cell migration and the production of amphiregulin and IL-12B growth factors by the bronchial epithelium, facilitating tissue repair [61,62]. Tryptase also possesses anti-inflammatory activity as it can cleave several pro-inflammatory cytokines and chemokines including eotaxin 1/3, CCL7, and IL-21, thereby inactivating them (Figure 2) [63,64].

Although chymase and tryptase are the best-known mast cell proteases, mast cell granules also contain carboxypeptidase A3 and cathepsin G [56,65]. The role of these two proteases in asthma has not yet been elucidated. However, recent studies have shown that carboxypeptidase A3 can cleave angiotensin and form angiotensin-(1-9), suggesting that the protease may contribute to vasodilation and immune cell infiltration in asthma [66], and cathepsin G has been shown to cleave the alarmins IL-18 and IL-33 as well as the stem cell factor (SCF), IL-3, IL −6, IL-7, IL-15, IL-31, and vascular endothelial growth factor (VEGF), suggesting a regulatory role in asthmatic inflammation (Figure 1) [67].

## Serotonin

Serotonin (5-Hydroxytryptamine) is a neurotransmitter commonly known for its psychological significance. However, it has been found to be elevated in the serum of asthma patients as one of the constituents released from mast cell granules upon IgE activation [68]. Studies in animal models have shown that serotonin alleviates airway inflammation [69]. In human cell culture models, serotonin stimulates macrophages and bronchial epithelium to produce the chemoattractant IL −8 by activating MAPK and ERK [70]. Serotonin may also contribute to structural changes by promoting the airway and vascular remodeling observed in asthma. Macrophages increase their production of transforming growth factor beta 1 (TGF-β1) in response to serotonin, and both chemicals promote bronchial smooth muscle proliferation [71]. Serotonin also promotes vasoconstriction and proliferation of pulmonary artery smooth muscle cells via regulation of Bcl-2 and BAX, resulting in arterial thickening [71,72]. Despite its pro-asthmatic influence, macrophages treated with serotonin show an increase in the expression of anti-inflammatory cytokines with a concomitant decrease in pro-inflammatory cytokines [73].

Another neurotransmitter released by mast cell granules is pituitary adenylate cyclase-activating polypeptide (PACAP). Mast cells express MRGPRX2 which acts as PACAP receptor and can trigger degranulation of mast cells [74]. PACAP also induces bronchial muscle relaxation, which has been demonstrated in human cells *in vitro* and in mice with asthma *in vivo* [75,76].

## Interleukins

Mast cells communicate with other cells and provide the link between innate and acquired immunity primarily by releasing cytokines, which can be divided into 3 major classes: Interleukins, chemokines, and growth factors.

Interleukins are a large family of secretory proteins that play a role in cell-to-cell communication by binding to surface receptors and stimulating various functions. When activated by FcεRI, mast cells release IL-1β, IL-6, IL-13, and TNF-α, which are involved in the pathology of asthma. TNF-α is elevated in the serum of asthma patients and correlates with poor ventilation. By interacting with its receptor on bronchial epithelial cells TNF-α induces them to express IL-1β, IL-8, IL-6, and CXCL10 by activating the NF-κB pathway, thereby promoting immune cell accumulation. TNF-α also increases cell proliferation, which is beneficial for wound healing but may also cause thickening of airway epithelium [77–79]. Asthma and allergies in the airways are usually associated with increased mucus production due to the activity of mast cells’ IL-1β and IL-13. IL-1β increases mucus release from the bronchial epithelium by upregulating the expression of Muc5ac through activation of the transcription factors hypoxia inducible factor-1 alpha (HIF-1α) and NF-κB [80], and exacerbates inflammation by promoting the differentiation of ILCs to ILC3 and enhancing their production of IL-17 which in turn stimulates epithelial cells to recruit neutrophils via IL-8 release. Consequently, both IL-1β and IL-17 levels correlate positively with inflammatory markers and inversely with lung function [81,82]. Airway epithelium expresses the IL-13 receptor (IL-13 Rα1), and their interaction regulates Muc5ac via signal transducer and activator of transcription 6 (STAT6) activation [36]. Other consequences of IL-13 signaling in the airways include synergizing with LTD4 and histamine by the upregulation of the leukotriene receptor CYSLTR1 and the histamine receptor H1R, increasing calcium uptake, leading to bronchial contraction [83]. Airway injury increases the epithelial expression of IL-13 Rα2, IL-13 signals the release of heparin-binding epidermal growth factor (HB-EGF) via this receptor, promoting airway repair and wound healing [36]. Another interleukin closely associated with inflammation is IL-6. Its levels are also elevated in the serum and sputum of asthma patients and inversely correlated with lung function [79,84]. IL-6 enhances the proliferation and maturation of mast cells and potentiates their response to FcεRI stimulation. The amplification of mast cell response to FcεRI stimulation is represented by the increased release of IL-8, GM-CSF, and upregulated airway remodeling gene signatures. IL-6 mediates these effects by enhancing the phosphorylation of PLCγ, STAT3, MAPK [85–88].

## Chemokines

Chemokines are another type of cytokines. Their main function is to stimulate cell recruitment by interacting with their respective cell surface receptors. Infiltration of immune cells is a major feature of asthma. Granulocytes, innate lymphoid cells, and macrophages are increased in the airways of asthma patients [89,90]. Mast cells play an essential role in chemotaxis by secreting a cocktail of chemokines in response to FcεRI cross-linking and by inducing the production of chemokines by surrounding cells [91,92]. Granulocytes are abundant in the airways and sputum of asthma patients; eosinophil increase airway hyperresponsiveness and their recruitment is mediated by CCL4 [89,93]. Neutrophils play an important role in defending the respiratory tract against pathogens by releasing reactive oxygen species and different proteases, however their activation is also suggested to contribute to airway inflammation during asthma. Neutrophil’s recruitment can be driven by CXCL1, IL −8, CCL2, or CCL7 released from mast cells [45,94]. IL-8 is a potent chemokine that also increases CD4^+^ helper T cells [95]. Although chemokines are primarily known for their chemotactic functions, they can also exert other effects. IL-8 was found to increase the proliferation of pulmonary fibroblasts. Another example is CCL2, in addition to neutrophil recruitment, CCL2 induces monocyte infiltration and proliferation through phosphoinositide 3-kinases (PI3K) and MAPK activation. The airway epithelium also expresses the CCL2 receptor CCR2B and their interaction induces mucus production through MAPK activation [94,96,97].

ILC1/2/3 are increased in the sputum of asthma patients [89]; the recruitment of ILC2 is mediated by CCL1, while their differentiation into ILC1 and ILC3 subtypes is facilitated by IL-1β, which is also produced by mast cells. ILC1 and ILC3 numbers in sputum samples from asthmatic patients were correlated with inflammatory macrophage subtype (M1), however, the number of ILC2 was correlated with anti-inflammatory macrophage subtype (M2), portraying the difference in functions of ILCs types [81,98]. Although ILCs mediate the differentiation of monocytes into specialized macrophage subtypes, mast cells are responsible for monocyte infiltration by secreting CCL3 and CCL5 [89,99,100]. Finally, airway smooth muscle cell migration has been suggested to contribute to airway remodeling in asthma. The airway smooth muscles of asthmatic patients show increased sensitivity to CXCL3 released from mast cells. Although under normal conditions CXCL3 interacts with CXCR2 and CXCR1 on smooth muscle cell’s surface and activates P38 and ERK1/2 MAPK signaling, in asthmatic cells CXCL3 signaling is more dependent on CXCR1 and PI3K signaling [101]. Thus, mast cells are not only directly involved in airway inflammation in asthma, but also attract various types of immune cells and cause structural changes.

## Growth factors

Airway remodeling is a common feature of asthma. It is manifested by changes in cell distribution, thickness, and vascularization of the respiratory tract. Various mast cell mediators contribute to tissue repair and remodeling of asthmatic airways [36,71,73]. These capabilities are essential for the healing of injured airway tissues; however, excessive growth and neovascularization can bedeterimental. Mast cells produce heparin-binding epidermal growth factor (HB-EGF),VEGF-A and VEGF-C [102–104]. HB-EGF promotes lung fibroblast proliferation and increases NF-κB activity, thereby increasing the expression of IL-8, which in turn increases fibroblast proliferation and migration, inducing airway remodeling [105]. Neovascularization in asthmatic airways is mediated by VEGF-A and VEGF-C. The former promotes angiogenesis – the formation of blood vessels – while the latter promotes lymphangiogenesis – the formation of lymphatic vessels. Increased vascularization is observed in asthma patients compared to healthy individuals. This is accompanied by a positive correlation between the density of microvasculature in the mucosa’s connective tissue and asthma’s severity; possibly providing additional pathways for immune cells to infiltrate bronchial tissues [106]. VEGF-A also induces mucus production of bronchial epithelial cells through interaction with the VEGFR2 receptor expressed on their surface thus activating Rho-kinase and upregulating Muc5Ac expression [107]. On the other hand, VEGF-C promotes the proliferation of lymphatic endothelial cells, their chemotaxis, and the formation of lymphatic tubes through VEGFR-3 signaling and increasing the expression of vascular cell adhesion molecule 1 (VCAM-1) [108]. VEGF-C is thought to play a protective role in asthma; a decrease in lymphatic vessels was observed in biopsies with fatal asthma, suggesting that they may be crucial for the drainage of mucosal edema from the asthmatic airways [109].

The GM-CSF is another cytokine secreted by mast cells. It was originally designated as a hematopoietic growth factor, but its functions go far beyond that (Figure 2). GM-CSF promotes the differentiation of eosinophils and basophils from hematopoietic progenitor cells. GM-CSF also has a number of effects on monocytes.It recruits monocytes by increasing their expression of chemokine surface receptors; and promotes their activation by upregulating their production of reactive oxygen species, and their expression of genes mediating endocytosis, antigen processing and presentation. GM-CSF also tilts monocytes differentiation toward the anti-inflammatory M2 subtype facilitating the repair of the epithelium as well as modulating inflammation by polarizing regulatory T-cells [110,111]. GM-CSF plays an important role not only in tissue repair but also in healing and maintainingairway barriers either directly by stabilizing junctions in the airway epithelium, or indirectly by activating macrophages and stimulating their release of growth factors [112–114].

## The role of mast cells in disease

Because they reside in tissues exposed to the external environment, mast cells are among the first immune cells to respond to bacterial and parasitic infections by recognizing molecular patterns associated with the pathogen via TLRs, cross-linking with parasites via IgE, or responding to internal stress signals released by infection via cytokine receptors, such as IL −33. Mast cells are essential for efficient clearance of pathogens. This was observed in mast cell-deficient mice whose immune responses to *Streptococcus agalactiae* were weakened, impairing clearance of bacteria. When mast cells are exposed to bacterial toxins, they recruit neutrophilic granulocytes and release pro-inflammatory mediators such as IL −6, TNF-α, and lipid mediators, thereby eliminating the bacteria and reducing bacterial colonization [5,8]. Moreover, the importance of mast cells in parasitic infections has also been demonstrated in *Strongyloides ratti*, where the mast cell deficient model showed impaired parasite defense and slowed response to the alarmin IL −33 [9]. Although mast cell activation is essential for the body’s defense against infection, inappropriate activation can lead to deleterious results. Allergy sufferers possess IgE antibodies to specific antigens, which can activate mast cells in response to allergens in their environment. Because mast cells are located in various body tissues, they contribute to various forms of allergic diseases, such as airway hyperresponsiveness, atopic dermatitis, and food allergies, in which mediators released by IgE-activated mast cells exacerbate inflammation [14]. Inappropriate mast cell activation is also described in individuals with mast cell activation syndrome (MCAS). After exclusion of other mast cell-related diseases such as allergies and mastocytosis, MCAS is characterized by episodes of symptoms caused by mast cell-released mediators, such as urticaria, wheezing, and nasal conjunctivitis; laboratory evidence of elevated markers of mast cell activation, particularly serum tryptase concentration; and resolution of symptoms in response to targeted therapies against mast cells and mediators [115]. Although the main causative agent of MCAS has not yet been identified, patients usually have multiple mutations in mast cell regulatory genes such as stem cell factor receptor (KIT), tyrosine kinase platelet-derived growth factor receptor α (PDGFRα), and H4R (HRH4) [116]. In addition, MCAS has been associated with the severity of COVID −19 and chronic disease after COVID −19. Mast cells contribute significantly to the cytokine storm of COVID −19, and mast cell-targeting drugs have been shown to be effective in controlling severity [117,118].

In addition, mast cells are important components of the tumor microenvironment, which is flooded with chemokines. Increased mast cell infiltration in tumors has been associated with poorer prognosis [119,120]. Tumor-infiltrating mast cells promote tumor growth through the release of IL −1β and blood microvessel formation through CXCL1 and VEGF-A; they promote cancer metastasis through the release of IL −8 and suppress T-cell immunity to cancer through the expression of programmed death-ligand 1 (PD -L1) [119–121]. However, mast cells sensitized to the oncogenic receptor HER2/neu successfully targeted breast cancer cells and induced their apoptosis through a TNF-α-dependent mechanism [122].

## Mast cell mediators as therapeutic targets

The role that mast cells play in allergic diseases makes them a desirable therapeutic target. One approach is to target the mediators released by mast cells. For example, an anti-tryptase antibody has been developed to target the effects of tryptase on airway structure and contraction and anaphylaxis in asthma [61]. In addition, the activity of mediators released by mast cells can also be blocked by receptor antagonists such as fevipiprant, the PGD2 receptor antagonist. Because PGD2 receptors are expressed on airway epithelium, smooth muscle, and various immune cells, blocking these receptors has produced favorable results in clinical trials by reducing asthma complications such as eosinophil infiltration and airway remodeling [123]. Another approach to combat mast cell-related disease is to target mast cell activation altogether. This can be achieved by IgE antibodies, one example being omalizumab, which is used in allergy and asthma. Omalizumab has also been tried in MCAS, where it effectively relieved patients’ symptoms [124]. Blocking mast cell activation to treat MCAS has also been demonstrated with tofacitinib. Janus kinases (JAKs) are involved in mast cell proliferation and degranulation. Blocking JAKs with tofacitinib has been reported to improve MCAS and asthma by reducing mast cell accumulation and pro-inflammatory mediator release [125,126].

## Conclusion

In asthma and allergic diseases, mast cells are stimulated by the allergen-IgE complex to release a variety of *de novo* synthesized and granularly stored mediators. These mediators released by mast cells are the main drivers of inflammation, as they include cytokines, lipid mediators, and proteases that can recruit other immune cells, disrupt airway structures, and promote airway remodeling. However, like the immune system as a whole, mast cells are a double-edged sword, as these mediators can also have protective and anti-inflammatory effects. Mast cells are essential for a rapid immune response against bacteria and parasites, while mast cell proteases are able to inactivate pro-inflammatory cytokines such as IL −6 and IL −33, while PGE2 released from mast cell granules can reduce degranulation. Recently, mast cells have received considerable attention due to their contribution to the COVID −19 cytokine storm and the potential protective properties of their mediators against this storm [118]. To elucidate this multifunctional role of mast cells in disease, an understanding of the causes and effects of the mediators released by mast cells is essential.

## Future perspective

Many aspects of the activity and therapeutic potential of mast cells remain unclear. This is in part because mast cells are heterogeneous cells, as evidenced by their granule content, surface receptors, and localization in health and disease. In addition to the different mast cell subtypes localized in different tissues, i.e., skin mast cells, mucosal mast cells, synovial mast cells, the different localization of MCTC and MCT subtypes in disease is also intriguing. Moreover, the different subtypes differ in their behavior, releasing different mediators under different conditions [43,127]. Understanding the effect of different stimulants on the different subtypes in the course of inflammatory diseases may be an important area of future research. With the advances in single cell transcriptome analysis, the heterogeneity of mast cells and their response to different stimulants can be further explored to find new targets for the treatment of inflammatory diseases. Existing therapeutics focus on blocking mast cell activation and inhibiting the activity of pro-inflammatory mediators. However, understanding the mechanisms and role of mast cell anti-inflammatory mediators could also lead to an alternative approach that would allow us to use these mediators and their analogs to treat inflammatory diseases.
